# Hepatitis C Virus Infection Trends in Italy

**Published:** 2012-01-20

**Authors:** Daniel Lavanchy

**Affiliations:** 1 World Health Organization, Geneve, Switzerland

**Keywords:** Hepatitis C, Infection, Italy

In previous issue of Hepatitis Monthly, La Torre G et al. from Sapienza University and the Catholic University of the Sacred Heart located in Rome, Italy, have published their findings on the incidence of hepatitis C virus (HCV) infections in Italy [1]. In the Italian general population, they estimate that the overall trend, and also the trends in gender and different age groups, has decreased significantly over the period from 1996 to 2006, with an overall significant decrease in incidence from 2.02 to 0.55 per 100,000. The incidence rates were estimated using the data from the system for acute viral hepatitis in Italy (SEIEVA) and population data from ISTAT. A marked decrease in incidence was observed in all age groups. A similar rate of decrease was observed in the 25-64-year-old and the greater than 65-year-old age groups. A more pronounced decrease was observed in the 15-24-year-old age group, whereas a low decrease was observed in the 0-14-year-old age group, primarily because of the existing low incidence in 1996 [1]. In the male population, which had a significantly higher incidence rate than the female population in 1996, the decrease was more marked with a clear trend of diminishing gender differences in HCV incidence in 2006, and with a lower (but probably not significant) incidence rate in males aged 0-14 years (0.05 vs. 0.15/100,000). The estimated decrease in the overall annual incidence was -12.5%. This study shows an encouraging favorable trend toward a low incidence of HCV in Italy, and it confirms previous findings in this country [2][3]. The trend described in this study is similar to the trends observed in Canada [4], Western Europe [4], and the USA [5][6][7]; however, contrary trends have been observed in Eastern Europe and Russia [4], and also among adolescents and young adults in some states of the USA [8][9][10] and in US population groups [11]. Estimation of the incidence of HCV infection remains difficult; reporting systems can underestimate the incidence of HCV infection despite well-established surveillance systems, such as that in Italy. Regular population-based surveys should be conducted to confirm these findings.

The notable decrease in the incidence rate seen in the last 2 decades in Italy is mainly attributed to the improvement in the safety of blood supplies and blood products, use of safe injection practices, and implementation of universal precautions in medical settings. Intravenous drug use, other parenteral exposure i.e. ear piercing, tattooing), surgery, hospitalization, and dental treatment remain common risk factors among patients with HCV infection, particularly in resource-limited settings. Therefore, there is still no room for complacency. The public health agenda warrants maintaining surveillance, prevention, and control of HCV infection. The joint-point regression method used in this study may help to assess the impact of public health policies to meet the challenges posed by HCV infection and disease and to foster new public health strategies. However, it must be kept in mind that infectious disease epidemics rarely follow linear trends over time.
